# The relationship between radiotherapy dose and cognitive outcomes in functional brain network hubs of glioma patients with predominantly left frontal lobe lesions

**DOI:** 10.1093/noajnl/vdaf163

**Published:** 2025-07-18

**Authors:** Laurien De Roeck, Rob Colaes, Patrick Dupont, Stefan Sunaert, Steven De Vleeschouwer, Paul M Clement, Charlotte Sleurs, Maarten Lambrecht

**Affiliations:** Department of Oncology, KU Leuven, Belgium; Department of Radiation-Oncology, University Hospitals Leuven, Belgium; Leuven Brain Institute, KU Leuven, Belgium; Department of Imaging & Pathology, KU Leuven; Department of Neurosciences, KU Leuven, Belgium; Leuven Brain Institute, KU Leuven, Belgium; Leuven Brain Institute, KU Leuven, Belgium; Department of Imaging & Pathology, KU Leuven; Department of Neurosurgery, University Hospitals Leuven, Belgium; Department of Neurosciences, KU Leuven, Belgium; Leuven Brain Institute, KU Leuven, Belgium; Department of General Medical Oncology, University Hospitals Leuven, Belgium; Department of Oncology, KU Leuven, Belgium; Department of Oncology, KU Leuven, Belgium; Department of Cognitive Neuropsychology, Tilburg University, Tilburg, The Netherlands; Leuven Brain Institute, KU Leuven, Belgium; Department of Oncology, KU Leuven, Belgium; Department of Radiation-Oncology, University Hospitals Leuven, Belgium

**Keywords:** advanced imaging, connectome, functional MRI, glioma, neurocognition

## Abstract

**Background:**

Emerging evidence suggests that cognitive dysfunction may result from damage to the brain’s functional network. This study explores the dose-dependent susceptibility of functional hubs to radiotherapy (RT) and associations with cognitive outcomes.

**Methods:**

Attention, language, memory, motor, and executive functioning were assessed ≥1-year post-radiotherapy in 39 WHO grade 2 or 3 glioma patients with lesions predominantly located in the left frontal lobe and 50 matched healthy controls. Using resting-state functional imaging, weighted functional graphs were constructed for each participant, identifying hubs through graph measures. Linear regression models and Spearman’s rho correlations assessed associations between mean RT dose per region and cognitive domains.

**Results:**

Higher RT doses to the left fusiform and inferior temporal gyri were linked to memory impairment (r(37) ≥ −0.565, *P*_FDR_ ≤ 0.026), while poorer language outcomes were associated with higher doses to the left pars opercularis, rostral middle frontal gyrus, and caudate (r(37) ≥ −0.510, *P*_FDR_ ≤ .040). Attention deficits were linked to higher doses to the left posterior cingulate, precentral, supramarginal, and postcentral gyrus ((37) ≥ −0.499, *P*_FDR_ ≤ .040), with the left postcentral gyrus also associated with executive dysfunction (r(37) = −0.526, *P*_FDR_ = .029). Significant correlations between RT dose and cognitive outcomes were more frequent in hubs than in non-hubs (50% vs. 12%, *P* = .005) and exclusively found in left-sided regions.

**Conclusions:**

RT seems to adversely affect left-sided functional hubs in a dose-dependent manner in glioma patients, which may contribute to cognitive dysfunction. Protecting these regions from the dose may potentially mitigate cognitive side effects in glioma patients. However, since most lesions were located in the left hemisphere and baseline testing was unavailable, a potential effect of tumor location cannot be entirely ruled out.

**Key Points**

- Cognitive impairment is prevalent in glioma patients post-RT.
- Higher dose to functional hubs was associated with poor cognitive performance.
- Functional hubs might serve as potential OARs for cognition.

**Importance of the Study**

With the current advances in radiotherapy for adult gliomas, such as proton therapy, we can significantly reduce radiation doses to healthy brain tissue while maximizing treatment efficacy. However, determining which healthy brain regions to spare in order to preserve cognitive functioning remains a topic of ongoing debate. Previously, cognitive side effects linked to individual brain regions have been inconsistent and lack established dose-volume parameters. This inconsistency may stem from the fact that cognitive functioning relies on the integration of information from various spatially distributed brain regions, rather than isolated components. In our study, we therefore investigated the dose–response relationship in functionally defined brain network hubs—highly interconnected network nodes—between RT dose and cognitive functioning. Our findings reveal that higher RT doses to left-sided network hubs were associated with poorer cognitive performance, and these associations were stronger than the association between hippocampal RT dose and memory.

Radiation therapy (RT) is an important treatment modality in the treatment of glial and other brain tumors, yet it is associated with long-term cognitive side effects.^1^ The mechanisms behind these cognitive impairments and the specific brain structures affected by the tumor or its treatment are poorly understood.^2–6^ Despite advances in RT techniques that allow for a more conformal dose distribution with selective sparing of specific brain regions, there is still uncertainty regarding which areas are most critical to spare from RT dose for preserving cognitive function. This uncertainty stems from inconsistent findings in studies focusing solely on specific anatomical brain regions,^7–9^ often overlooking the complex interplay and information exchange between different brain regions.

When conceptualizing the brain as a network, where information flows between brain regions or nodes is facilitated through edges, certain nodes—termed hubs—emerge as pivotal brain structures for cognition due to their central role in the brain’s network.^10–13^ Similar to major train stations that handle a higher volume of passengers and connections, these hubs appear to manage a disproportionate amount of information flow,^14^ and can be classified into 2 types based on their interconnecting edges.^15^ Structural hubs are brain regions with extensive connections through white matter tracts, typically identified using diffusion-weighted imaging, while functional hubs serve as central relay points that coordinate neural activity across various brain regions, with connectivity often assessed using functional magnetic resonance imaging (fMRI).^16,17^

This disproportional amount of information flow throughout these hubs is characterized by a high level of glucose consumption, neuronal firing, metabolism and gene expression related to ATP production and plasticity.^18^ This heightened metabolic demands therefore renders them more vulnerable to neurotoxic events.

In our previous research, we observed that structural network hubs are sensitive to radiation dose, with higher doses to these brain regions linked to cognitive dysfunction. Similarly, functional network hubs may be vulnerable to the neurotoxic effects of radiotherapy, resulting in cognitive impairments.

Given that both functional and structural network hubs have been linked to cognition,^10,11^ the next question that arises is how these functional and structural hubs are interconnected within the brain network, as functional connectivity (FC) is likely facilitated by structural pathways to support the ongoing information transfer between these regions. Although the coupling of structural and FC across the entire brain has been associated with cognition,^19–21^ the specific interconnections between functional and structural network hubs, as well as the relative importance of hub-hub versus hub-nonhub connections in cognition, remain relatively unexplored.^22,23^

This study aims to explore the sensitivity of functional network hubs to RT and the implications of radiation-induced hub damage for cognitive functioning. Furthermore, we will examine the connectivity patterns between structural and functional hubs in both impaired and non-impaired individuals, aiming to determine the relative importance of hub-hub versus (non)hub-nonhub connections in maintaining cognitive function. Understanding these dynamics will provide deeper insights into the neurobiological underpinnings of RT-induced cognitive side effects and help identify critical brain regions or connections that should be protected from RT dose during radiation treatment.

## Materials and Methods

### Study Design and Patients

This cross-sectional study enrolled adult patients (≥18 years) with WHO grade 2 or 3 gliomas (2016 WHO classification), who underwent RT at the University Hospitals of Leuven, Belgium, between 2007 and 2019. Participants were excluded if they had other brain tumors (eg, meningioma, metastases), a prior psychiatric diagnosis, a relapse following (chemo)radiotherapy, or MRI contraindications.

Additionally, a healthy control group matched for sex, age, and educational level was included. Data collection for all participants occurred between 2021 and 2022. All participants completed a cognitive test battery, a self-report inventory, and underwent advanced neuroimaging on the same day.

The study received ethical approval from the Ethical Committee of the University Hospitals of Leuven (S63580). Written informed consent was obtained from all participants.

### Cognitive Assessments

Various cognitive domains were evaluated using a one-hour battery of cognitive tests: the Hopkins Verbal Learning Test-Revised (HVLT-R) to assess memory, the Grooved Pegboard test for motor functioning, the Controlled Oral Word Association Test (COWAT) for language, and several subtests, including the Trail Making Test parts A & B,^24^ the digit span and digit symbol substitution tests from the Wechsler Adult Intelligence Scale (WAIS IV),^25^ and the Stroop Color and Word Test^26^ for executive functioning and attention. Cognitive domains were determined according to inter-test correlations and the DSM-V definitions (Table S1).^27^

Raw scores from these cognitive assessments were converted into w-scores, which were adjusted for age and education based on the healthy control group (details in Supplementary Materials).^28^ Cognitive impairment was defined as instructed by the International Cancer and Cognition Task Force guidelines, as having 2 or more test scores with w-scores at or below −1.5 or at least one test score with a w-score at or below −2.0.^29^

### Image Acquisition, Network Construction, and Hub Identification

Magnetic resonance (MR) images were obtained using a 3T Philips Achieva scanner, which included 3D FLAIR, T2-, T1-weighted images (MPRAGE), diffusion-weighted images (DWI), and resting-state functional MR scans. Details regarding the acquisition and preprocessing steps of all sequences can be found in the Supplementary Materials.

Semi-automatic brain lesion segmentation was conducted following the methodology described previously,^11^ using a custom script that combined resseg, HD-glio-auto, and FastSurfer.^30–32^ This process was followed by manual correction to ensure accuracy.

On the T1-weighted images, 78 network nodes were defined by parcellating the cortex and subcortical brain regions (caudate, thalamus, putamen, amygdala, pallidum, accumbens area, and hippocampus) according to the Desikan-Killiany-Tourville atlas^33^ using Fastsurfer (v2.0).^32^

The functional and structural connections or the edges interconnecting the 78 network nodes were modeled based on resting-state functional MR scans and DWI, respectively.

From the resting-state functional MRI scans, FC matrices were generated for each patient by computing partial correlations between the time series of each pair of nodes (78 × 78). Absolute values of these correlations were used in the functional adjacency matrix. To mitigate the impact of compromised connections in tumor-affected areas, connectivity values between regions overlapping more than 50% with the tumor and unaffected areas were set to zero.

From the DWI, structural connectomes were constructed as described previously.^11^

Weighted graph measures were then calculated from the resultant graphs (see Supplementary Materials for detailed methods).

Functional and structural network hubs were identified in the healthy control group based on the hubscore as defined by Van den Heuvel^14^ (Supplementary Materials). Nodes with a hubscore of zero in more than 50% of healthy controls were classified as functional non-hubs.

### Radiotherapy Treatment and Dose Calculation

Radiotherapy treatment sum plans were extracted from Varian Eclipse treatment planning software and registered to the parcellated T1-weighted images. The mean administered dose to each node, including both functional hubs and non-hubs, was calculated using FSL^34^ and MRtrix3.^35^ Nodes with at least 50% overlap with the tumor lesion were excluded from the analysis to minimize the influence of tumor infiltration.

### Statistical Analysis

As previously reported, the functional network of glioma patients showed increased segregation, indicated by higher clustering coefficient values.^36^ We therefore hypothesize that higher local radiotherapy (RT) dosage might alter the functional brain network, resulting in greater segregation and subsequently poorer cognitive outcomes.

To test this hypothesis, we first employed a linear regression model to predict the clustering coefficient values for each hub and non-hub based on the received RT dose. Next, we explored the associations between RT dosage and cognitive outcomes by correlating the RT dose per node with the w-scores for each cognitive domain (*n* = 5) using Spearman correlation coefficients. To determine if these correlations were more common in hubs compared to non-hubs, we used McNemar’s test.

The significance threshold for statistical tests was set at α < 0.05, and false discovery rate correction was applied to account for multiple comparisons.

To explore the potential impact of (1) the irradiated volume and (2) mean dose over all nodes, (3) tumor size and (4) tumor location on cognitive function, we conducted partial correlation analyses between RT dose and cognitive outcomes, while controlling for (1) planned target volume (PTV) and (2) mean RT dose, (3) tumor size and (4) tumor location over all nodes, respectively.

Subsequent analyses examined differences in both structural and functional connectivity (edges) between either patients and controls or impaired and non-impaired patients. Mann–Whitney *U* tests were employed to compare connectivity involving structural and functional hubs (hub-hub connections), structural hubs versus non-hubs ([non]hub-nonhub connections), and functional hubs versus non-hubs ([non]hub-nonhub connections). A significance level of *P* < .05 (uncorrected) was used to identify statistically significant differences in these exploratory analyses. Additionally, a Pearson Chi-Square test was conducted to assess whether significant differences in edges occurred more frequently in either structural or functional connectivity between the groups. All statistical analyses were performed using SPSS (version 29.0.2.0).

## Results

### Patients

Patient characteristics have been previously detailed and summarized in Table S2.^37^ In brief, the study included 39 patients, with a median age of 35 years (range: 18–57 years), while the control group consisted of 50 healthy individuals, with a median age of 40 years (range: 21–72 years). The most common diagnosis was WHO grade 2 oligodendroglioma (51%), with lesions predominantly located in the left hemisphere (64%) and the frontal lobe (74%). All participants underwent photon radiotherapy (3DCRT vs. VMAT), with total doses between 54 and 60 Gy, delivered in fractions of 1.8 to 2.0 Gy. The median planning target volume (PTV) was 250 mm³ (range: 58–658 mm³). The mean time between radiotherapy and cognitive testing was 5 years, standard deviation of 3 years and a range between 1 and 14 years. At the time of cognitive evaluation, 66% of patients were using antiepileptic drugs. Among these, the majority were on monotherapy (67%; 13 out of 16 patients used levetiracetam), while a smaller proportion were on dual therapy (31%; 4 out of 8 used levetiracetam) or triple therapy (7%; all patients used levetiracetam).

### Cognitive Performance

As previously described, a substantial proportion of patients (69%) exhibited cognitive impairment in at least one domain.^37^ Specifically, memory impairment was present in 43.6%, motor dysfunction in 51.3% of patients, attention deficits in 35.9%, impaired verbal fluency in 35.9%, and executive dysfunction in 30.8%. Cognitive assessments were conducted, on average, 5 years after RT. No statistically significant differences were found in the prevalence of domain-specific cognitive impairments between patients treated with 3D-conformal RT and those treated with VMAT RT techniques.

### Associations Between RT Dose and Clustering Coefficient

Among healthy controls, 8 nodes were identified as functional network hubs (Figure S1) and 41 out of 78 nodes as functional non-hubs (Supplementary Table S3).

Although the RT dose was associated with the clustering coefficient values in 4 non-hub nodes, the statistical significance did not hold after false discovery rate correction. Specifically, higher RT doses to the left precentral gyrus and the right lateral occipital gyrus were associated with lower clustering coefficient values. Conversely, higher RT doses to the right anterior cingulate gyrus and the postcentral gyrus were associated with higher clustering coefficient values (Table S4).

### Associations Between RT Dose and Cognitive Functioning

**Figure 1.**
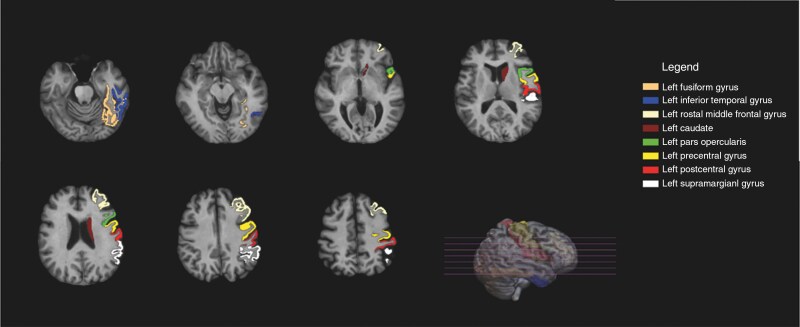
Functional hubs and non-hubs with the most significant dose–response relation on cognitive performance (*P* ≤ .001)

Patients who received higher average RT doses to 9 out of 78 nodes showed significantly worse cognitive performance (Table 1 and Figure 1). Specifically, increased doses to the left fusiform gyrus and inferior temporal gyrus were significantly correlated with memory impairment (r(37) = −0.57, *P*_FDR_ = .026; r(37) = −0.60, *P*_FDR_ = .025). Higher doses to the left pars opercularis (frontal lobe; r(37) = −0.55, *P*_FDR_ = .026), rostral middle frontal gyrus (r(37) = −0.55, *P*_FDR_ = .029), and caudate (r(37) = −0.51, *P*_FDR_ = .040) were strongly associated with poorer language outcomes. Additionally, higher RT doses to the left posterior cingulate (r(37) = −0.50, *P*_FDR_ = .040), precentral gyrus (r(37) = −0.52, *P*_FDR_ = .035), supramarginal gyrus (r(37) = −0.54, *P*_FDR_. = 027), and postcentral gyrus (r(37) = −0.55, *P*_FDR_ = .026) were significantly linked to attention deficits. Moreover, higher doses to the left postcentral gyrus were also associated with executive dysfunction (r(37) = −0.53, *P*_FDR_ = .029).

**Table 1.** Significant Correlations of RT Mean Dose and Cognitive Performance (w-Scores) Per Node

Node group	Node	Cognitive domain	Spearman corr.	P-uncorr	p_FDR_	Mean dose (Gy; median, range)
Hub	Left fusiform gyrus	Memory	−0.57	< .001	.026	13.27 (0.16–50.63)
	Left inferior temporal gyrus	Memory	−0.60	<.001	.025	11.96 (0.40–49.67)
	Left pars opercularis	Language	−0.55	.001	.026	27.21 (0.06–56.94)
	Left postcentral gyrus	Executive function	−0.53	.001	.029	16.49 (0.20–56.94)
		Attention	−0.55	<.001	.026	
Non-hub	Left posterior cingulate gyrus	Attention	−0.50	.002	.040	11.45 (0.05–56.08)
	Left precentral gyrus	Attention	−0.52	.001	.035	18.58 (0.24–52.07)
	Left rostral middle frontal gyrus	Language	−0.55	.001	.029	20.34 (0.05–58.10)
	Left supramarginal gyrus	Attention	−0.54	.001	.027	9.75 (0.01–57.97)
	Left caudate	Language	−0.51	.001	.040	32.41(0.09–59.33)
	Left hippocampus	Memory	−0.48	.003	.059	21.83 (0.70–59.79)

These correlations exceeded those observed between memory performance and mean RT doses to the left hippocampus (r(37) = −0.48, *P*_FDR_ = .059) and right hippocampus (r(37) = −0.06, *P*_FDR_ = .895).

Overall, significant correlations between RT dose and cognitive outcomes were significantly more frequent in hub regions compared to non-hub regions (50% vs. 12%, *P* = .005) and were exclusively found in left-sided brain regions.

Post-hoc analyses confirmed consistent significant associations between RT dose and cognitive outcomes in all above-mentioned brain regions, even when controlling for planning target volume (PTV), the mean RT dose across all nodes (Table S5), tumor location, and tumor size (Table S6).

### Functional and Structural Hub Interconnections and the Link With Cognition

**Figure 2.**
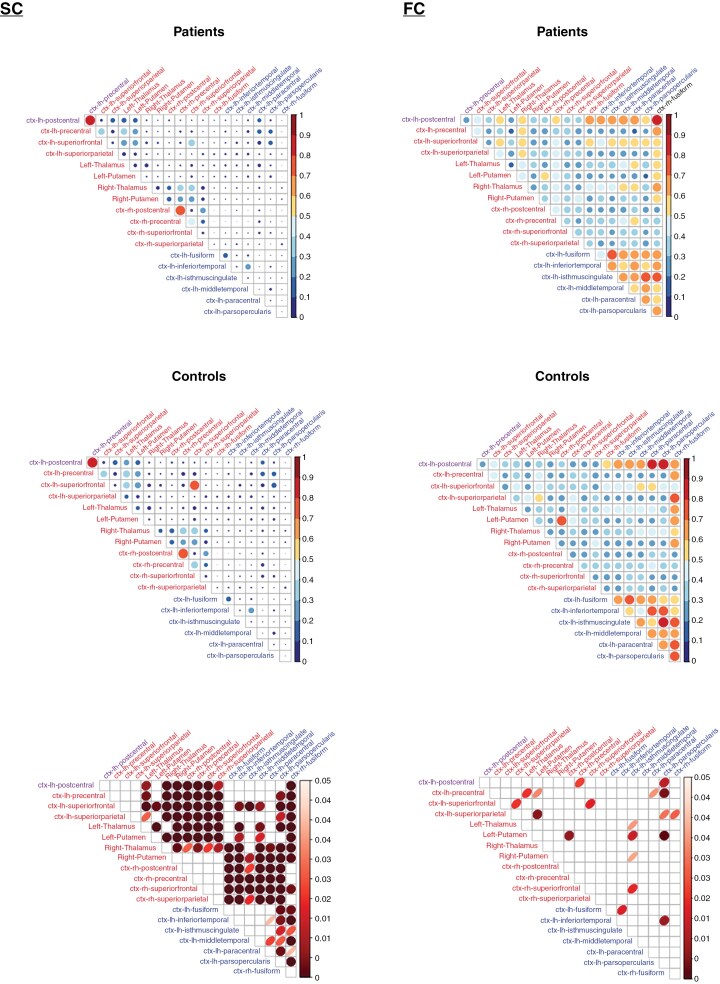
Correlograms for structural connectivity (SC) and functional connectivity (FC) between structural hubs and functional hubs (hub-hub connections) in glioma patients versus healthy controls with associated uncorrected *P*-value maps (Mann–Whitney *U* tests) Correlograms displaying the structural connectivity (SC, first row), represented by the number of streamlines, and functional connectivity (FC, second row) between structural hubs (red) and functional hubs (blue) in patients versus controls. The left postcentral gyrus is highlighted in purple as it serves as both a structural and functional hub. The color scale indicates SC/FC values, with the highest values in red and the lowest in blue. The size of the circles also reflects SC/FC values, with larger circles representing higher values. On the right, p-value maps are shown, where p-values were obtained through Mann–Whitney U tests comparing SC/FC between impaired and non-impaired patients. Only significant values are displayed.

Functional hubs seemed to exhibit stronger functional interconnections in both patients and controls compared to structural hubs (Figure 2). However, significant differences in hub-hub connections between patients and controls were significantly more frequent in structural connectivity compared to functional connectivity (64% vs. 11%, χ² = 430.72, 95% CI = 0.851–0.925, df = 1, two-sided *P* < .001).

**Figure 3.**
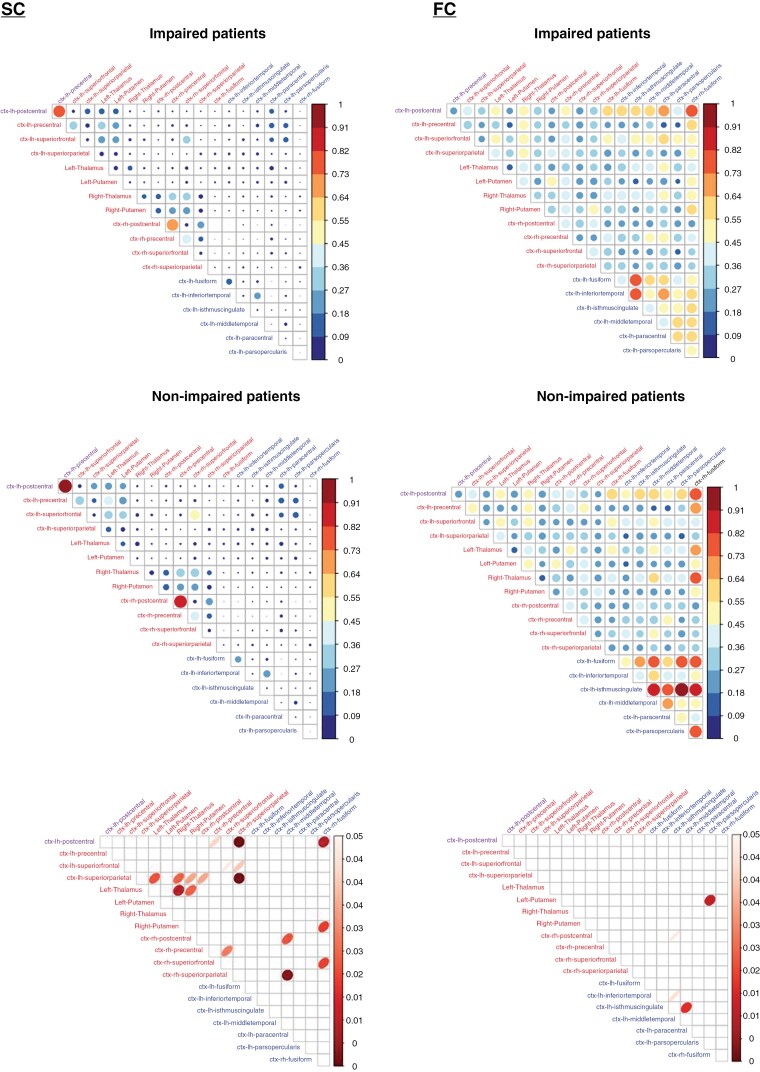
Correlograms for structural connectivity (SC) and functional connectivity (FC) between structural hubs and functional hubs (hub-hub connections) in impaired vs unimpaired patients with associated p-value maps (Mann–Whitney *U* tests) Correlograms displaying the structural connectivity (SC, first row), represented by the number of streamlines, and functional connectivity (FC, second row) between structural hubs (red) and functional hubs (blue) in impaired versus non-impaired patients. The left postcentral gyrus is highlighted in purple as it serves as both a structural and functional hub. The color scale indicates SC/FC values, with the highest values in red and the lowest in blue. The size of the circles also reflects SC/FC values, with larger circles representing higher values. On the right, p-value maps are shown, where p-values were obtained through Mann–Whitney U tests comparing SC/FC between impaired and non-impaired patients. Only significant values are displayed.

We further investigated differences in hub-hub connections between cognitively impaired and non-impaired patients (Figure 3). Functional hubs appeared to exhibit more coherent and stronger interconnections, particularly in non-impaired patients. Nevertheless, significant differences in structural hub-hub connections were significantly more prevalent compared to functional hub-hub connections (10% vs. 2%, χ² = 15.854, df = 1, 95% CI = 0.037–0.114, two-sided *P* < .001). Importantly, both types of structural hub-hub connections—between structural hubs themselves and between structural hubs and functional hubs—appeared to play significant roles.

Given the apparent role of structural hub-hub connections, we also compared their relative importance with structural (non)hub-nonhub connections in impaired versus non-impaired patients. However, there was no significant difference in the number of significantly different structural hub-hub connections (Figure 3) compared to structural (non)hub-nonhub connections (Figure S3) in impaired versus non-impaired patients (10% vs. 7%, χ² = 3.2344, df = 1, 95% CI = −0.006–0.065, two-sided *P* = .072).

## Discussion

Technological advances in cranial irradiation cannot reach their full potential to preserve higher-order cognitive functions without a comprehensive understanding of the brain’s anatomical-functional organization. We hypothesized that functionally highly interconnected regions (functional hubs) play a significant role in cognitive functioning and can be impacted by RT dose. We observed that higher RT doses to 9 specific left-sided brain nodes were significantly correlated with worse cognitive performance, particularly affecting memory, language, and attention. Consistent with our hypothesis, correlations between RT dose and cognitive outcomes were more frequently observed in functional hub regions compared to functional non-hub regions. These associations remained significant even after controlling for PTV and mean RT dose across all nodes. Our findings corroborate previous studies, which found that damage to hubs leads to more widespread cognitive deficits, compared to lesions to non-hub regions.^38^ Importantly, our study adds value by exploring for the first time the dose-dependent effects of radiation-induced damage to functional hubs.

One potential mechanism for this hub-specific damage in response to radiation treatment may involve critical damage to hubs, causing them to fail and necessitating a redistribution of network traffic to other brain areas.^39^ The most interconnected hubs then bear the largest share of the load, and as these hubs fail, traffic shifts to secondary hubs, potentially triggering a cascade of failure and network reorganization, ultimately impacting cognitive function. Previous research has shown that radiation can induce changes in dendritic length and complexity, as well as reductions in synaptic protein levels, suggesting that this hub-specific damage could be driven by modifications in neuronal structure and synaptic plasticity.^40^

In addition to observing significantly more correlations in hubs, all associations between RT dose and cognitive outcomes were detected exclusively in left-sided functional hubs, suggesting a role of lateralization in cognitive function. This finding aligns with our previous research in the same cohort, which identified associations between radiotherapy dose and cognitive performance, particularly in left-sided structural hubs,^37^ and with other studies showing patients with left-sided tumors to exhibit more pronounced cognitive deficits compared to those with right-sided tumors.^4,41–43^ This lateralization effect was also evident for the hippocampus. In line with the studies by Haldbo-Classen et al. and Goda et al.,^43,44^ we found a (nearly) significant relationship between the dose to the left hippocampus and verbal memory. However, no such correlation was observed for the right hippocampus. Importantly, the association between verbal memory and the left hippocampal dose was relatively weak compared to the relationships we identified between cognition and doses to other brain regions implicated in FC, such as the left postcentral gyrus, precentral gyrus, supramarginal gyrus, fusiform gyrus, inferior temporal gyrus, rostral middle frontal gyrus, caudate, and pars opercularis (frontal lobe). However, due to the absence of baseline cognitive data, a comprehensive understanding of the causal relationship between radiotherapy and cognitive outcomes remains unattainable. Furthermore, as the majority of tumors were located in the left hemisphere, the potential influence of tumor location or infiltration cannot be entirely excluded. Consequently, we recommend that future research efforts seek to validate these findings through longitudinal data collection, incorporating multiple relevant covariates to more accurately reflect the complex nature of cognitive functioning.

Although we previously found the network of glioma survivors to exhibit increased network segregation (ie, higher clustering coefficient values),^36^ this effect did not show a significant linear relationship with RT dose. Potentially, the tumor’s influence on network segregation may overshadow that of radiation, contributing to increased clustering coefficient values in the network of patients irrespective of the RT dose. Another contributing factor could be individual variability, where genetic differences, overall brain health, and pre-existing conditions lead to diverse responses to RT, obscuring clear patterns in network properties. Additionally, threshold effects may be at play, suggesting that changes in clustering coefficient values might only manifest beyond specific radiation dose thresholds. Below these thresholds, compensatory mechanisms within the brain may maintain the overall network integrity.

Given the importance of functional and structural hubs in cognition,^11^ we further investigated the relative impact of hub-hub connections compared to (non)hub-nonhub connections on cognitive functioning across different types of connectivity. Our study demonstrates that structural and functional hubs are not identical but are highly interconnected, suggesting that some functional connections between hub regions likely reflect direct structural connections. Despite functional hubs exhibiting greater interconnectivity, the number of significantly different functional hub-hub connections was lower compared to structural hub-hub connections.

The markedly altered structural hub-hub connectivity, and to a lesser extent functional hub-hub connectivity, observed in cognitively impaired patients aligns with the network failure theory. With an average post-therapy duration of 5 years, most patients were in the chronic phase of cascading network failure, characterized by prolonged and excessive FC leading to structural brain damage.^39^

In non-impaired patients, the maintained hub-hub connectivity may indicate that the overall connectome has not yet collapsed, providing a degree of resilience, especially in patients with favorable molecular subtypes^22^

However, the lack of significant difference between structural hub-hub and structural (non)hub-nonhub connections implies that this damage extends beyond hubs into the chronic phase of cascading failure, suggesting that impairment of (non)hub-nonhub connections could also contribute to the cognitive dysfunction observed in these patients.

The study’s findings should be interpreted taking several limitations into consideration. First, its cross-sectional design lacks baseline (pre-RT) measurements of neurocognitive function, which restricts the ability to fully comprehend the causal impact of radiotherapy on cognitive outcomes. Moreover, our control group consisted of healthy volunteers rather than (matched) glioma patients who underwent surgery but did not receive radiation, which would have provided a more appropriate comparison to assess the impact of surgery on the cognitive functions evaluated in our study.

Second, cognition is influenced by multiple factors beyond radiotherapy, including tumor volume, grade, edema, use of anti-epileptic drugs, and mutations in IDH genes.^45–47^ Future studies with larger sample sizes and longitudinal data collection should comprehensively investigate these variables.

Third, it’s notable that a majority of glioma patients in our study had left frontal lesions, resulting in higher radiation doses to these regions. While radiation doses did vary widely across brain nodes, potential bias from this regional effect cannot be entirely ruled out.

Fourth, we excluded nodes with more than 50% lesion overlap to minimize potential confounding effects of tumor location. This cutoff was selected to strike a balance between maintaining statistical power (by retaining a sufficient number of nodes) and reducing the impact of tumors on our results. However, choosing a cutoff does always involve some degree of subjectivity. To verify the robustness of our results, we repeated the analysis with 2 different cutoffs, being 30% and 70%, showing identical results. Finally, we compared structural and FC between hub regions in patients versus controls and impaired versus unimpaired patients. However, the strength (ie, weight) of the connections is dependent on the definition of these weights (ie, number of streamlines for structural connectomes and partial correlations for functional connectomes), and can therefore differ across studies.

Despite its limitations, the study possesses several strengths. We conducted a thorough exploration of imaging, cognitive, and dose-volume parameters in a relatively rare patient cohort, supported by a robust sample size. Moreover, our findings were reinforced by the inclusion of a control group matched for sex, age, and education, which served to mitigate external influences. Additionally, we utilized cognitive assessments endorsed by international organizations (ICCTF/EORTC),^29,48^ allowing for evaluation across a wide range of cognitive domains. Standardized testing was uniformly administered by the same examiner, and imaging was performed on a single scanner.

Furthermore, to minimize interobserver variability, we utilized automated delineation of the structures under investigation. Lastly, we implemented advanced techniques for constructing connectomes and rigorously corrected our statistical analyses for multiple testing, with the exception of the exploratory analysis on the significance of hub-hub connections.

Understanding which brain regions are crucial for cognition could potentially influence RT treatment plans by adjusting parameters such as radiation dose to these specific functional hubs. This approach aims to preserve cognitive functioning in patients undergoing RT treatment. Given that the functional importance of these hubs and the brain’s adaptability may change over time, assessing FC could also inform decisions on the timing of radiation treatment. In selected patients with low-grade gliomas—particularly when a functional hub is located near the tumor or when plasticity appears limited—it has been hypothesized that delaying radiotherapy could allow time for functional reorganization. Such a delay might be supported by the use of IDH inhibitors, such as vorasidenib. ^49^However, postponing radiotherapy carries the risk of tumor progression into critical functional hubs, which could lead to greater cognitive impairment. As such, this approach should be pursued with caution and requires further investigation.

## Conclusion

Our study in glioma patients with lesions predominantly located in the left frontal lobe shows associations between higher RT doses to functional brain hubs and cognitive outcomes, potentially due to their susceptibility to dose-dependent damage from radiotherapy. Insights into the complex network dynamics of the brain in cognitive function and dysfunction post-radiotherapy hold potential for refining treatment strategies. When validated in longitudinal studies, sparing these specific hubs and their interconnected pathways might be a way to mitigate cognitive decline and improve patient outcomes in neuro-oncological care.

## Supplementary Material

vdaf163_suppl_Supplementary_Materials_1

## Data Availability

The data that support the findings of this study are available from the corresponding author upon reasonable request. The code is available on the Open Science Framework (link).
