# Supplementary material for: The relationship between radiotherapy dose and cognitive outcomes in functional brain network hubs of glioma patients with predominantly left frontal lobe lesions
Source: Neurooncol Adv. 2025 Jul 18;7(1):vdaf163. doi: 10.1093/noajnl/vdaf163 (PMC12365900; doi:10.1093/noajnl/vdaf163)
Supplement: vdaf163_suppl_Supplementary_Materials_1 [file vdaf163_suppl_supplementary_materials_1.docx]

**Supplementary Materials**

**Methods**

*Study design & participants*

Patients were screened based on a medical database. All WHO grade 2 and grade 3 glioma patients in routine clinical follow-up with a WHO performance status of 0 to 2 were eligible. To exclude acute therapy-effects, we only included patients who had completed therapy at least one year before inclusion (surgery and/or chemoradiotherapy).

Since the aim of this specific study is to assess the dose response effect of radiotherapy, we solely included irradiated patients.

Healthy controls were recruited via online forums and were matched at individual level for age (maximal age difference of 3 years), sex and education (levels of Verhage).

All participants completed a self-report inventory, a cognitive test battery and neuroimaging protocol, completed on the same day.

*Cognitive data*

Raw cognitive assessment scores were transformed into w-scores, which are analogous to z-scores but adjusted for specific covariate(s) to account for the large variability in the sample, i.e. age and education in this study. Based on a linear regression model, age- and education specific (ISCED 2011 definition) regression coefficients were calculated based on the healthy controls dataset and obtained test scores were then subtracted from the estimated scores**.** [1]

As longer response times indicate worse performance, w-scores for time-critical tests were inverted (multiplied by -1) for consistency in interpretation (i.e. higher w-score indicates better performance). Subsequently, these test scores were categorized into six main cognitive domains (Table S1) based on the DSM-V definition of neurocognitive functioning and between-test correlations**.** [2] The domain summary scores were calculated for all participants by averaging the test-specific w-scores of that particular domain**.** [3]

*Image acquisition*

Magnetic resonance (MR) images were acquired on a Philips Achieva scanner operating at 3T with a 32-channel phased-array head coil. First, T1-weighted images (MPRAGE) were acquired (7 minutes, voxel size=0.80x0.80x0.80 mm^3^ isotropically, FA=8◦, TR/TE=5.8/2.5 ms, FOV=320 × 320 voxels, 208 slices), followed by a 4-minute 3D FLAIR scan (voxel=1x1x1 mm^3^ isotropically, TI= 165 ms, FA=90◦, TR/TE=4800/340 ms, NSA=2, FOV=256 × 256 voxels, 183 slices).

Resting-state functional magnetic resonance images were acquired during a 8-minute session, during which participants were instructed to keep still with their eyes closed, refrain from engaging in specific thoughts, and relax without falling asleep (EPI, voxel size=2.5mm3 isotropically, FA=70°, TR/TE= 1000/33 ms, FOV = 240 × 240 × 146 mm, reconstruction matrix = 96 × 96 × 56, 450 volumes, multiband width=4).

Multi-shell diffusion-weighted images were acquired with two phase encoding directions = AP (8 minutes) and then PA (8minutes), voxel size = 2x2x2mm^3^, FA = 90°, TR/TE = 5000/80ms, FOV = 112 x 112x 72 voxels, b-values = 0/700/1200/2800 s/mm² with a total of 10/24/40/76 gradient directions respectively, SENSE=2.5, and multiband=2.

*Image preprocessing*

Image processing was performed using matlab-based scripts (Matlab R2023a and Bash) and validated toolboxes.

First, semi-automatic lesion segmentation of resection cavity, tumoral tissue and/or perilesional gliosis, and ventricles was performed by using resseg [4]**,** HD-glio-auto [5] and FastSurfer [6]**,** respectively. Subsequently, all lesions were manually corrected in ITK-snap (v3.6.0)[7] by a radiation-oncologist (LDR). Lesion-free and T1-weighted images and masks were created using Virtual Brain Grafting (VBG v0.61[8]). The VBG-corrected anatomical scan was then used to parcellate the cortex and subcortical regions using FastSurfer (v2.0[6]), according to the Desikan-Killiany-Tourville (DKT) atlas which resulted in 78 cortical parcellations**.**[9, 10]

Functional MR images were preprocessed using fMRIPrep 23.0.[53], including re-alignment, slice-time correction, field unwarping, bias field correction, and spatial normalization to ICBM152 2009c nonlinear asymmetrical template. Subsequently, data were further processed using the CONN‐toolbox (CON 20b,[54]). This included functional outlier detection (based on scrubbing of motion‐affected functional volumes). We addressed potential sources of spurious variance by regressing out the realignment and scrubbing parameters, as well as signals from the white matter and ventricular system. To further minimize the impact of low-frequency drift and high-frequency physiological noise, we applied linear detrending and temporal band-pass filtering (0.009–0.8 Hz).

DWI preprocessing included Gibbs-ringing artifact removal[11] and denoising[12] using MRtrix3 (v3.0,[13]), eddy current, EPI distortion and motion correction[14] using FSL (v6.0.1) Eddy, and N4 bias-field correction[15] using ANTs[16].

*Structural connectome construction*

An affine transformation followed by a nonlinear registration (mutual information cost-function) was done using ANTs (v2.3.5[16]) to transform the parcellations to the mean fiber orientation distribution (FOD) image**.**

For each subject, tissue-specific constrained spherical deconvolution (CSD) response functions and whole-brain tractograms (iFOD2, ten million fibres, dynamic seeding), were constructed using multi-shell multi-tissue CSD, and anatomically-constrained tractography with SIFT2 re-weighting of the streamlines in MRtrix3 (v3.0[13]). [17, 18]

*Graph measure calculations and definitions*

Weighted graph measures (based on this weighted graph) of nodal strength, average path length, betweenness centrality and clustering coefficient were calculated for all 78 nodes for the structural and functional graphs separately.

Since graph measures depend on the weight distribution, we normalized the graph measures: 1000 random equivalent graphs (i.e. with the same number of nodes and the same weight distribution) were generated by randomly permuting the edges, while taking into account the self-connections (no weight) and the connectome symmetry and removing graphs with floating nodes. Next, the graph measures were divided by the corresponding median values obtained from the random networks in order to scale the observed measures relative to what is expected in random networks with the same weight distribution. Graph measures were calculated using the Brain Connectivity toolbox (v2019-03-03) and an in-house developed MATLAB script. We opted to include these specific graph measures as nodal strength, clustering coefficient, betweenness centrality, and shortest path length are essential for calculating the hubscore [19].

In brief, the shortest path length between two nodes is the path with minimal cost. It provides insights into the overall efficiency of information or signal propagation in a network and was calculated by the algorithm of Dijkstra. Cost was defined as the inverse of the connection weight.

Clustering coefficient measures the degree of local clustering or connectivity within a network. It quantifies how well-connected the neighbors of a particular node are to each other.

Nodal strength is a measure of the cumulative influence or importance of a node within a network, taking into account the strength of its connections to other nodes. Nodes with higher nodal strengths are considered more central or influential within the network because they have stronger interactions with other nodes.

Betweenness centrality measures how often a node occurs on the shortest paths between any two other nodes, making it a key metric for identifying nodes that play a crucial role in facilitating information flow.

*Hub and non-hub definitions*

An overall hubscore for each node was calculated based on four nodal metrics: betweenness centrality, shortest path length, clustering coefficient, and nodal strength.[19] Nodes that ranked in the top 20% for betweenness centrality and nodal strength, and the bottom 20% for shortest path length and clustering coefficient, were awarded one point for each criterion met, resulting in a hubscore ranging from 0 to 4. Nodes achieving a total score of 2 or more in over 50% of subjects in each group were classified as network hubs.[19] A non-hub was defined as a node with a hubscore of zero in over 80% of healthy controls.

**References**

1. Rijnen SJM, Meskal I, Emons WHM, et al (2020) Evaluation of Normative Data of a Widely Used Computerized Neuropsychological Battery: Applicability and Effects of Sociodemographic Variables in a Dutch Sample. Assessment 27:. https://doi.org/10.1177/1073191117727346

2. Sachdev PS, Blacker D, Blazer DG, et al (2014) Classifying neurocognitive disorders: The DSM-5 approach. Nat Rev Neurol 10

3. Andrade C (2021) Z Scores, Standard Scores, and Composite Test Scores Explained. Indian J Psychol Med 43:. https://doi.org/10.1177/02537176211046525

4. Pérez-García F, Dorent R, Rizzi M, et al (2021) A self-supervised learning strategy for postoperative brain cavity segmentation simulating resections. Int J Comput Assist Radiol Surg 16:. https://doi.org/10.1007/s11548-021-02420-2

5. Kickingereder P, Isensee F, Tursunova I, et al (2019) Automated quantitative tumour response assessment of MRI in neuro-oncology with artificial neural networks: a multicentre, retrospective study. Lancet Oncol 20:. https://doi.org/10.1016/S1470-2045(19)30098-1

6. Henschel L, Conjeti S, Estrada S, et al (2020) FastSurfer - A fast and accurate deep learning based neuroimaging pipeline. Neuroimage 219:. https://doi.org/10.1016/j.neuroimage.2020.117012

7. Yushkevich PA, Pashchinskiy A, Oguz I, et al (2019) User-Guided Segmentation of Multi-modality Medical Imaging Datasets with ITK-SNAP. Neuroinformatics 17:. https://doi.org/10.1007/s12021-018-9385-x

8. Radwan AM, Emsell L, Blommaert J, et al (2021) Virtual brain grafting: Enabling whole brain parcellation in the presence of large lesions. Neuroimage 229:. https://doi.org/10.1016/j.neuroimage.2021.117731

9. Klein A, Tourville J (2012) 101 labeled brain images and a consistent human cortical labeling protocol. Front Neurosci. https://doi.org/10.3389/fnins.2012.00171

10. Desikan RS, Ségonne F, Fischl B, et al (2006) An automated labeling system for subdividing the human cerebral cortex on MRI scans into gyral based regions of interest. Neuroimage 31:. https://doi.org/10.1016/j.neuroimage.2006.01.021

11. Kellner E, Dhital B, Kiselev VG, Reisert M (2016) Gibbs-ringing artifact removal based on local subvoxel-shifts. Magn Reson Med 76:. https://doi.org/10.1002/mrm.26054

12. Veraart J, Novikov DS, Christiaens D, et al (2016) Denoising of diffusion MRI using random matrix theory. Neuroimage 142:. https://doi.org/10.1016/j.neuroimage.2016.08.016

13. Tournier JD, Smith R, Raffelt D, et al (2019) MRtrix3: A fast, flexible and open software framework for medical image processing and visualisation. Neuroimage 202

14. Andersson JLR, Skare S, Ashburner J (2003) How to correct susceptibility distortions in spin-echo echo-planar images: Application to diffusion tensor imaging. Neuroimage 20:. https://doi.org/10.1016/S1053-8119(03)00336-7

15. Tustison NJ, Avants BB, Cook PA, et al (2010) N4ITK: Improved N3 bias correction. IEEE Trans Med Imaging 29:. https://doi.org/10.1109/TMI.2010.2046908

16. Avants BB, Tustison NJ, Song G, et al (2011) A reproducible evaluation of ANTs similarity metric performance in brain image registration. Neuroimage 54:. https://doi.org/10.1016/j.neuroimage.2010.09.025

17. Smith RE, Tournier JD, Calamante F, Connelly A (2015) The effects of SIFT on the reproducibility and biological accuracy of the structural connectome. Neuroimage 104:. https://doi.org/10.1016/j.neuroimage.2014.10.004

18. Smith RE, Tournier JD, Calamante F, Connelly A (2012) Anatomically-constrained tractography: Improved diffusion MRI streamlines tractography through effective use of anatomical information. Neuroimage 62:. https://doi.org/10.1016/j.neuroimage.2012.06.005

19. Van Den Heuvel MP, Mandl RCW, Stam CJ, et al (2010) Aberrant frontal and temporal complex network structure in schizophrenia: A graph theoretical analysis. Journal of Neuroscience 30:. https://doi.org/10.1523/JNEUROSCI.2874-10.2010

**Supplementary Tables**

**Table S1 Cognitive (sub)tests grouped per cognitive domain**

| Cognitive domain | Neurocognitive test | Outcome measurement |
| --- | --- | --- |
| Memory | HVLT-R immediate recall | Sum score - learning |
|  | HVLT-R delayed recall | Sum score |
|  | HVLT-R recognition | Good recognition-mistakes |
| Executive functioning | TMT B | time |
|  | SCWT interference | Interference score |
|  | WAIS IV digit span backwards | Total number of series |
|  | WAIS IV sequencing | Total number of series |
| Attention / processing speed | WAIS IV symbol substitution | Sum score |
|  | TMT A | time |
|  | SCWT colors | time |
|  | SCWT words | time |
|  | WAIS IV digit span forward | Total number of series |
| Motor function | Grooved pegboard | Time (non)dominant hand |
| Language | COWAT semantic | Sum of words |
|  | COWAT phonemic | Sum of words |

HVLT-R: Hopkins Verbal Learning Test Revised; TMT: Trail Making Test; SCWT: Stroop Color Word Test; WAIS IV: Wechsler Adult Intelligence Scale, fourth edition; COWAT: Controlled Oral Word Association Test.

**Table S2 Descriptive characteristics of the participants**

| **Characteristics** | **Patients (n=39)** | **Healthy controls (n=50)** |
| --- | --- | --- |
| **Demographics** |  |  |
| Age at diagnosis (in years) |  |  |
| *Mean* (SD) | 36,97 (11,99) | 42.42 (13) |
| *Median* (range) | 35 (18-57) | 40 (21-72) |
| Sex: females, *n* (%) | 21 (54) | 25 (50) |
| Handedness |  |  |
| Right, *n (%)* | 32 (82) | 47 (94) |
| Left, *n (%)* | 13 (10) | 3 (6) |
| Both, *n (%)* | 2 (8) | 0 (0) |
| Anti-epileptic drug use |  |  |
| Yes, *n (%)* | 26 (67) | 0 (0) |
| Monotherapy | 16 (62) | 0 (0) |
| Dual therapy | 8 (31) | 0 (0) |
| Triple therapy | 2 (7) | 0 (0) |
| **Tumor treatment** |  |  |
| Time since radiotherapy in years, *mean* (SD) | 5,46 (3,36) |  |
| Surgery, *n* (%) | 32 (82) |  |
| Total excision | 16 (41) |  |
| Subtotal excision | 16 (41) |  |
| Biopsy only | 7(18) |  |
| Radiotherapy | 39 (100) |  |
| Total dose, *n*  (%) |  |  |
| 54 Gy | 20 (51) |  |
| 59.4 Gy | 7(18) |  |
| 60 Gy | 12 (31) |  |
| Technique, *n*  (%) |  |  |
| 3DCRT | 18 (46.2) |  |
| VMAT | 21 (53.8) |  |
| Chemotherapy | 35 (90) |  |
| PCV, *n* (%) | 23 (59) |  |
| TMZ, *n* (%) | 12 (31) |  |
| **Tumor location*** |  |  |
| Frontal, *n*  (%) | 29 (74) |  |
| Parietal, *n*  (%) | 5 (13) |  |
| Temporal, *n*  (%) | 11 (28) |  |
| Occipital, *n*  (%) | 1 (3) |  |
| Brainstem, *n*  (%) | 2 (5) |  |
| Thalamus, *n*  (%) | 1 (3) |  |
| Involved hemisphere |  |  |
| Left, *n*  (%) | 25 (64) |  |
| Right, *n*  (%) | 13 (33) |  |
| Both, *n*  (%) | 1 (3) |  |
| **PTV volume (mm^3^)** |  |  |
| Median (range) | 250 (58 -658) |  |
| **Tumor characteristics** |  |  |
| **Histology (WHO 2016)** |  |  |
| Oligodendroglioma, *n*  (%) | 20 (51) |  |
| Diffuse astrocytoma, *n*  (%) | 15 (39) |  |
| Anaplastic astrocytoma, *n*  (%) | 2 (5) |  |
| Anaplastic oligodendroglioma, *n*  (%) | 2 (5) |  |
| **WHO grade** |  |  |
| 2, *n* (%) | 29 (74) |  |
| 3, *n* (%) | 10 (26) |  |
| **IDH mutation** |  |  |
| IDH1-mutation, *n* (%) | 26 (67) |  |
| IDH1- wild type, *n* (%) | 8 (21) |  |
| IDH1, NOS, *n* (%) | 5 (12) |  |

* Overlap of multiple brain tumor locations possible. Abbreviations: Gy= Gray, 3DCRT= 3D-conformal radiotherapy, VMAT= Volumetric Modulated Arc Therapy, PCV= procarbazine, lomustine (CCNU) and vincristine, TMZ= temozolomide, WHO= World Health Organization, IDH= Isocitrate dehydrogenase, NOS= not otherwise specified.

**Table S3 Overview of the functional hubs and non-hubs defined on the hubscore in the healthy control group**

| ***Hubs*** | ***Non-hubs*** |
| --- | --- |
| Left fusiform gyrus | Left lateral occipital gyrus |
| Left inferior temporal gyrus | Left pars triangularis |
| Left isthmus of cingulate cortex | Left pericalcarine |
| Left middle temporal gyrus | Left posterior cingulate |
| Left paracentral gyrus | Left precentral gyrus |
| Left pars opercularis | Left rostral anterior cingulate gyrus |
| Left postcentral gyrus | Left rostral middle frontal gyrus |
| Right fusiform gyrus | Left superior parietal gyrus |
|  | Left supramarginal gyrus |
|  | Left insula |
|  | Left cerebellar cortex |
|  | Left caudate |
|  | Left putamen |
|  | Left pallidum |
|  | Left hippocampus |
|  | Left amygdala |
|  | Left accumbens area |
|  | Right caudate |
|  | Right putamen |
|  | Right pallidum |
|  | Right hippocampus |
|  | Right amygdala |
|  | Right cuneus |
|  | Right entorhinal cortex |
|  | Right inferior parietal gyrus |
|  | Right inferior temporal gyrus |
|  | Right lateral occipital gyrus |
|  | Right lateral orbitofrontal gyrus |
|  | Right medial orbitofrontal gyrus |
|  | Right parahippocampal gyrus |
|  | Right pars opercularis |
|  | Right pars orbitalis |
|  | Right pericalcarine |
|  | Right postcentral gyrus |
|  | Right posterior cingulate gyrus |
|  | Right precuneus |
|  | Right rostral anterior cingulate gyrus |
|  | Right superior frontal gyrus |
|  | Right superior parietal gyrus |
|  | Right superior temporal gyrus |
|  | Right transverse temporal |

*Ctx=cortex; lh=left; rh=right*

**Table S4 Linear regression results of impact RT dose on nodal clustering coefficient values**

| **Node** | **Node group** | **df** | **df (error)** | **F-value** | **p-uncorr** | **p_FDR_** | **t-value** | **β** |
| --- | --- | --- | --- | --- | --- | --- | --- | --- |
| Left precentral gyrus | Non-hub | 1 | 34 | 4.52 | .041 | .46 | -2.13 | -.34 |
| Right lateral occipital gyrus | Non-hub | 1 | 35 | 5.15 | .030 | .46 | -2.27 | .-36 |
| Right postcentral gyrus | Non-hub | 1 | 35 | 4.41 | .043 | .46 | 2.10 | .33 |
| Right rostral ant. cingulate gyrus | Non-hub | 1 | 34 | 5.66 | .023 | .44 | 2.38 | .37 |

**Table S5 Partial correlations of radiation mean dose and cognitive performance (w-scores) per node while controlling for radiation mean dose over all nodes or planned target volume (PTV)**

| **Node group** | **Node** | **Cognitive domain** | **Partial corr. controlling for overall mean dose(p-uncorr)** | **Partial corr. controlling for PTV (p-uncorr)** |
| --- | --- | --- | --- | --- |
| Hub | Left fusiform | Memory | -.55(.001) | -.45(.006) |
|  | Left inferior temporal | Memory | -.55(<.001) | -.46(.005) |
|  | Left pars opercularis | Language | -.52(.001) | -.50(.002) |
|  | Left postcentral | Executive function | -.39(.020) | -.45(.005) |
|  |  | Attention | -.49(.003) | -.50(.002) |
| Non-hub | Left posterior cingulate | Attention | -.42(.012) | -.46(.005) |
|  | Left precentral | Attention | -.46(.005) | -.49(.003) |
|  | Left rostral middle frontal | Language | -.51(.003) | -.48(.003) |
|  | Left supramarginal | Attention | -.47(.004) | -.52(.001) |
|  | Left caudate | Language | -.53(.001) | -.47(.005) |
|  |  |  |  |  |
|  | Left hippocampus | Memory | -.42(.012) | -.39(.021) |

**Table S6 Partial correlations of radiation mean dose and cognitive performance (w-scores) per node while controlling for tumor location or tumor size**

| **Node group** | **Node** | **Cognitive domain** | **Partial corr. controlling for tumor location (p-uncorr)** | **Partial corr. controlling for tumor size (p-uncorr)** |
| --- | --- | --- | --- | --- |
| Hub | Left fusiform | Memory | -.49(.003) | -.45(.006) |
|  | Left inferior temporal | Memory | -.55(<.001) | -.42(.010) |
|  | Left pars opercularis | Language | -.49(.003) | -.45(.006) |
|  | Left postcentral | Executive function | -.39(.020) | -.45(.005) |
|  |  | Attention | -.49(.003) | -.50(.002) |
| Non-hub | Left posterior cingulate | Attention | -.35(.025) | -.46(.005) |
|  | Left precentral | Attention | -.42(.010) | -.49(.003) |
|  | Left rostral middle frontal | Language | -.49(.003) | -.42(.010) |
|  | Left supramarginal | Attention | -.42(.010) | -.52(.001) |
|  | Left caudate | Language | -.39(.020) | -.39(.020) |
|  |  |  |  |  |
|  | Left hippocampus | Memory | -.40(.015) | -.35(.025) |

**Supplementary Figures**

**Figure S1: Functional hubs identified in healthy controls (hubscore ≥2)**


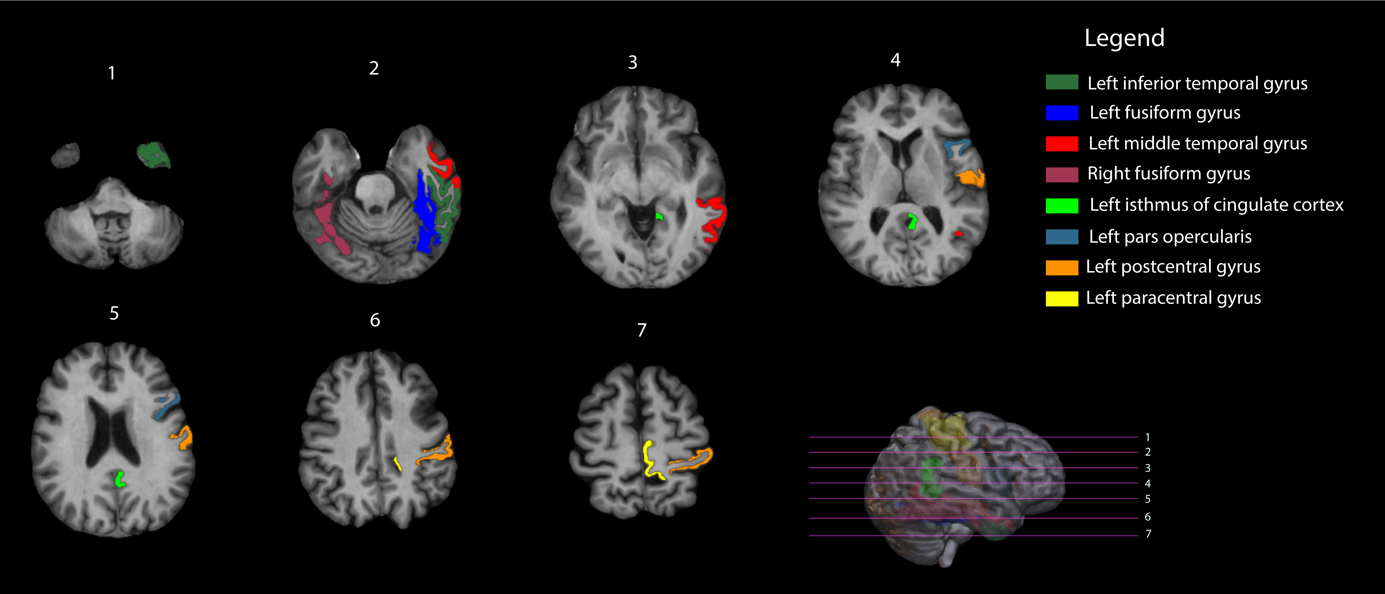


**Figure S2: structural connectivity (SC) and functional connectivity (FC) between functional hubs (blue) and non-hubs (magenta) to assess the relative importance of hub-nonhub connections**

**SC Impaired Patients Non-impaired Patients**

**
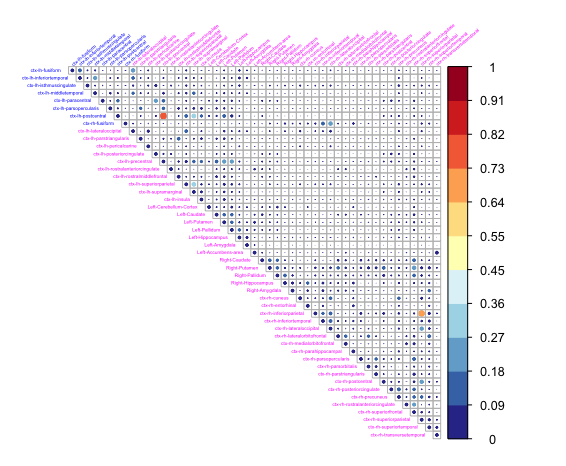

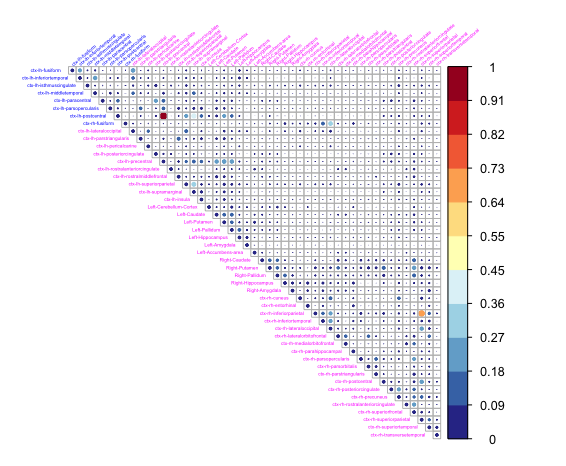

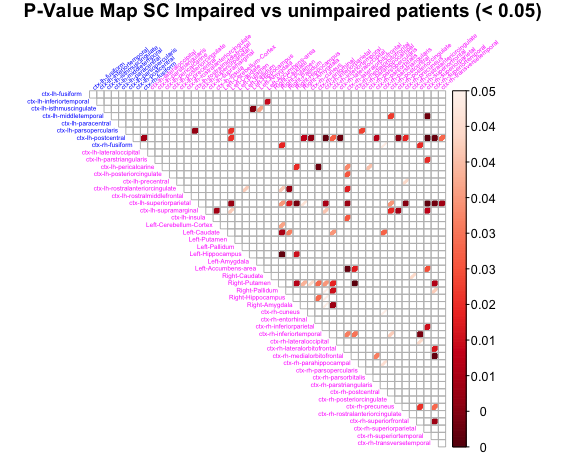
**

**FC Impaired Patients Non-impaired Patients**

**
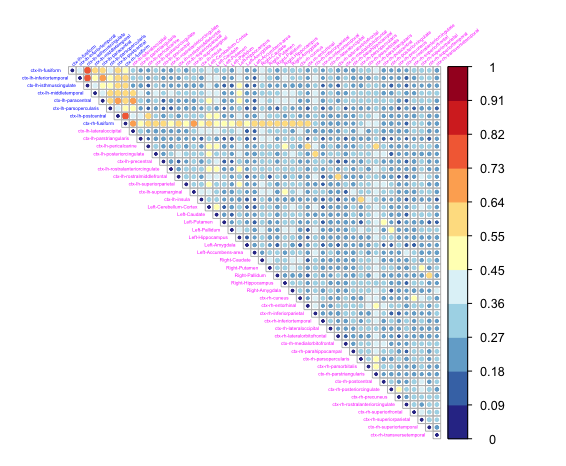

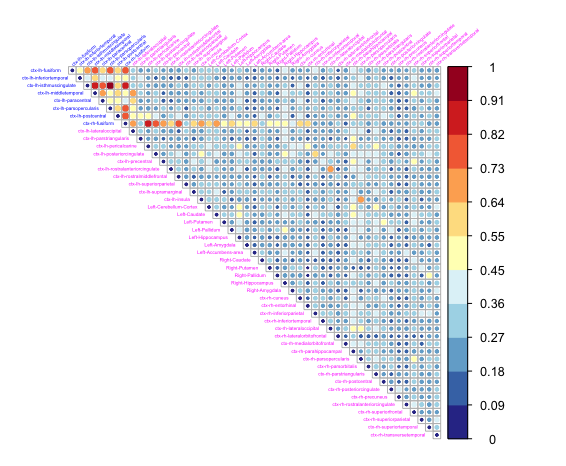

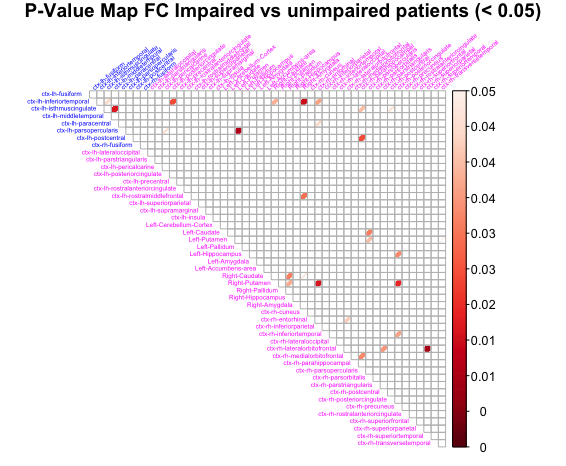
**

*Correlograms displaying the structural connectivity (SC, first row), represented by the number of streamlines, and functional connectivity (FC, second row) between functional hubs (blue)) and functional non-hubs (magenta). The color scale indicates SC/FC values, with the highest values in red and the lowest in blue. The size of the circles also reflects SC/FC values, with larger circles representing higher values. On the right, p-value maps are shown, where p-values were obtained through Mann-Whitney U tests comparing SC/FC between impaired and non-impaired patients. Only significant values are displayed.*

**Figure S3: structural connectivity (SC) and functional connectivity (FC) between structural hubs (red) and non-hubs (green) to assess the relative importance of hub-nonhub connections**

**SC Impaired Patients Non-impaired Patients**

**
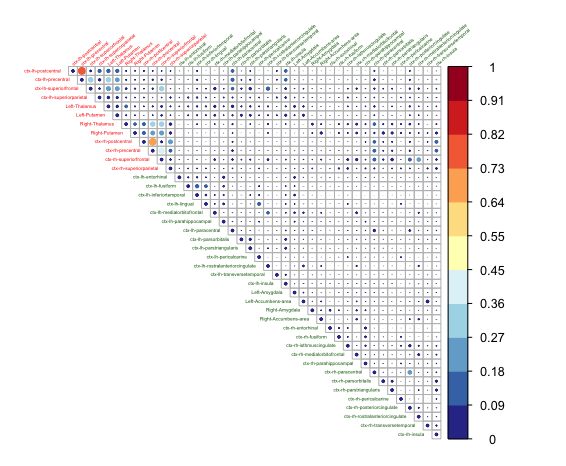

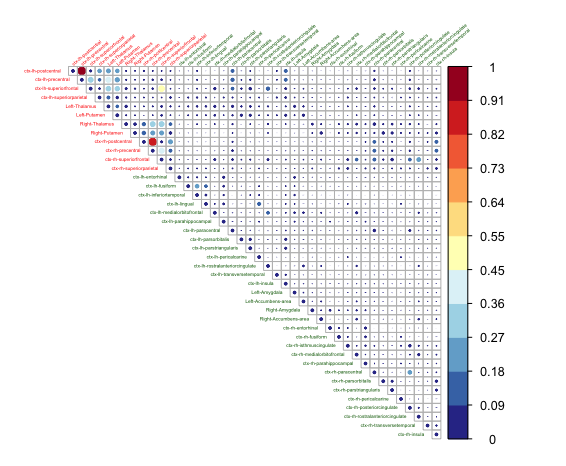

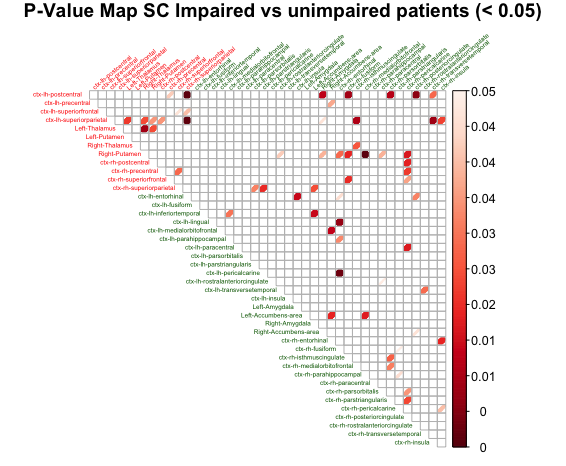
**

**FC Impaired Patients Non-impaired Patients**


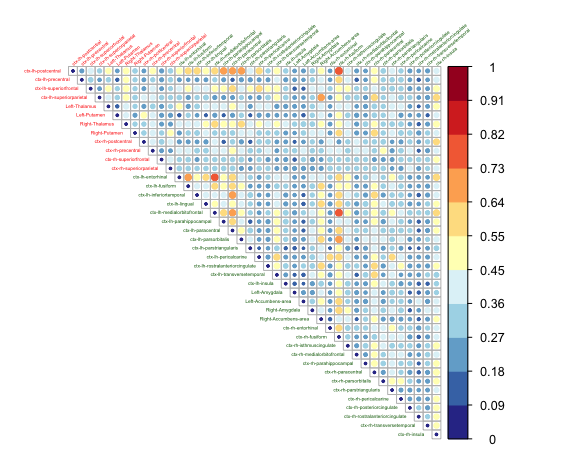

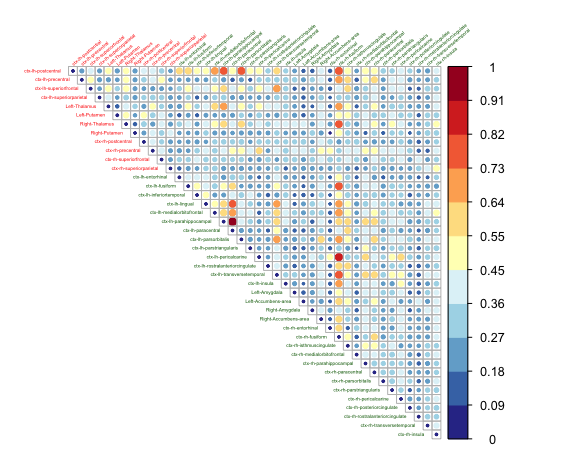

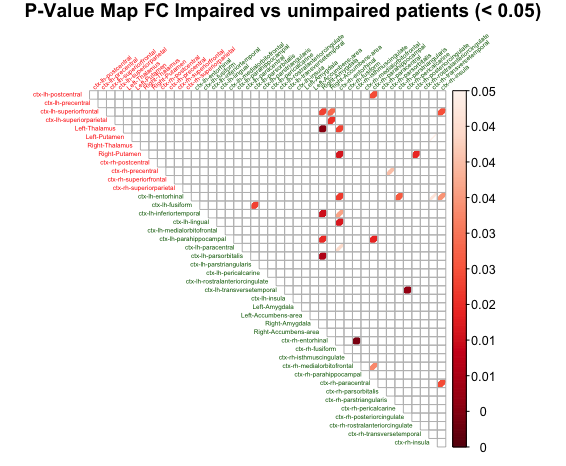


*Correlograms displaying the structural connectivity (SC, first row), represented by the number of streamlines, and functional connectivity (FC, second row) between structural hubs (blue) and structural non-hubs (magenta). The color scale indicates SC/FC values, with the highest values in red and the lowest in blue. The size of the circles also reflects SC/FC values, with larger circles representing higher values. On the right, p-value maps are shown, where p-values were obtained through Mann-Whitney U tests comparing SC/FC between impaired and non-impaired patients. Only significant values are displayed.*
